# Physiological relevance and generation-level stability of artificial intelligence-generated race-day warm-up and cool-down plans for sprint swimming: an expert-rated comparison of four large language models

**DOI:** 10.3389/fphys.2026.1923231

**Published:** 2026-08-27

**Authors:** TianYue Zhao, YiYao Peng, ZiXin Chen, Xiaojun Wang

**Affiliations:** 1 School of Physical Education, Sichuan University, Chengdu, Sichuan, China; 2 School of Law, Sichuan University, Chengdu, Sichuan, China

**Keywords:** artificial intelligence, content quality, exercise physiology, expert evaluation, generation stability, large language models

## Abstract

**Background:**

Large language model (LLM)-based artificial intelligence (AI) tools are increasingly used to generate sport and exercise training content. However, the content quality, sport-specific relevance, safety, and repeated-generation stability of AI-generated race-day plans remain insufficiently examined, particularly in competitive swimming. It also remains unclear how different AI tools may support coaches in drafting, comparing, and refining competition-day preparation plans.

**Objective:**

This study aimed to compare the expert-rated physiological relevance, content quality, and generation-level stability of race-day warm-up and immediate post-race cool-down plans generated by four publicly accessible LLM-based AI tools for a simulated 100-m freestyle swimmer, and to identify how these tools may serve as coach-supervised drafting and comparison aids in swimming race-day preparation.

**Methods:**

A simulation-based content-analysis design with a within-rater repeated-measures structure was used. Four AI tools—ChatGPT, Gemini, DeepSeek, and Doubao—each generated five independent outputs using the same standardized detailed prompt. Each output included a race-day warm-up plan and an immediate post-race cool-down plan. After anonymization and randomization, 32 experts with experience in coaching or teaching 100-m sprint swimming independently rated the plans using a 40-item multidimensional rating instrument. Inter-rater reliability was assessed using intraclass correlation coefficients and Krippendorff’s alpha. The primary analysis used a cumulative link mixed model with fixed effects for AI model, section, and their interaction, and random intercepts for rater, item, and generation ID. Exploratory domain-level and generation-level stability analyses were also conducted.

**Results:**

ChatGPT showed the highest descriptive overall quality. The cumulative link mixed model showed a significant model × section interaction (p <.001), indicating that model differences varied between the warm-up and cool-down sections. ChatGPT received higher ratings than DeepSeek and Doubao in the warm-up section and than all three other models in the cool-down section. Exploratory domain-level analyses showed differences across all 10 content-quality domains. Generation-level stability did not correspond directly to expert-rated quality: Doubao was the most stable model, whereas ChatGPT showed the most favorable overall quality profile but greater repeated-generation variability.

**Conclusions:**

Publicly accessible LLM-based AI tools can generate structured swimming race-day warm-up and immediate post-race cool-down plans, but their ability to translate sprint-swimming physiological principles into practical prescriptions differs across tools and plan sections. In this simulated 100-m freestyle scenario, ChatGPT showed the most favorable overall expert-rated profile, whereas Doubao produced more stable but lower-rated outputs. These findings suggest that AI-generated swimming race-day plans should not be used as stand-alone prescriptions. Instead, they may support coaches by providing preliminary drafts, alternative plan structures, and clearer prescription details for further expert screening and revision. Final decisions on intensity, sequencing, recovery, risk management, and individual adaptation should remain under qualified coach supervision.

## 1 Introduction

Large language models (LLMs), a major form of generative artificial intelligence (AI), are increasingly being used to generate sport and exercise training plans because they can rapidly convert general knowledge into practical, user-facing recommendations. However, existing evidence shows that AI-generated plans are not consistently optimal in scientific quality, safety, individualization, or practical applicability, and expert oversight remains necessary (Dergaa et al., 2024; Düking et al., 2024; Genç et al., 2025; Havers et al., 2025b; Puce et al., 2026; Yang et al., 2026b). More broadly, recent research in adjacent professional-content domains has shown that AI-generated outputs should not be judged by fluency alone, but also by deeper quality criteria such as structural integrity, logical coherence, readability, accuracy, and safety (Wilhelm et al., 2023; Zheng et al., 2025; Yang et al., 2026a). These concerns are relevant to sport settings because AI-generated plans may appear well organized while still lacking sufficient domain specificity, contextual adaptation, or evidence-based detail (Wilhelm et al., 2023; Dergaa et al., 2024; Düking et al., 2024; Genç et al., 2025; Havers et al., 2025b; Puce et al., 2026; Yang et al., 2026a; Yang et al., 2026b). Beyond content quality, the use of generative AI in sport also raises ethical concerns related to responsibility for AI-assisted decisions, data privacy, and potential linguistic or cultural bias, reinforcing the need for transparent and professionally supervised use (Lederman et al., 2025).

In competitive swimming, this issue is especially important because race-day preparation is highly event-specific, and warm-up design can influence subsequent performance, particularly in the transition period between pool warm-up and race start (Fradkin et al., 2010; West et al., 2013; Neiva et al., 2014a; McGowan et al., 2015; McGowan et al., 2016; McGowan et al., 2017; Ramos-Campo et al., 2020; Cuenca-Fernández et al., 2022; McKenzie et al., 2022). Warm-up is not merely a generic preparatory activity, but a performance-relevant process involving physiological activation, neuromuscular readiness, and event-specific technical preparation (Neiva et al., 2014a; McGowan et al., 2015). In sprint events such as the 100-m freestyle, this balance is especially delicate, because the athlete must achieve sufficient readiness without accumulating unnecessary fatigue (Neiva et al., 2014a; Neiva et al., 2014b; McGowan et al., 2015). Previous studies have shown that 100-m freestyle performance may be influenced by whether a warm-up is performed at all, by the type and volume of warm-up, by whether the stimulation is more race-pace or aerobic in nature, and by the duration of passive rest during the post-warm-up transition period (Balilionis et al., 2012; West et al., 2013; Neiva et al., 2014b; Neiva et al., 2017a; Neiva et al., 2017b; Czelusniak et al., 2021).

Recent swimming-specific work has further suggested that the transition phase itself deserves careful attention. Dryland re-warm-up, passive heating, hypoxic re-warm-up, and post-activation potentiation strategies have all been explored as ways to preserve readiness when delays occur between the pool warm-up and the race start. Although some of these strategies appear promising, findings are not fully consistent across studies or outcome measures, indicating that race-day warm-up design in sprint swimming remains a context-sensitive task rather than a fixed formula (Balilionis et al., 2012; West et al., 2013; Neiva et al., 2014b; McGowan et al., 2015; McGowan et al., 2016; Neiva et al., 2017a; Neiva et al., 2017b; Abbes et al., 2018; Ramos-Campo et al., 2020; Czelusniak et al., 2021; Cuenca-Fernández et al., 2022; McKenzie et al., 2022). This is practically important because 100-m freestyle performance is strongly shaped by phase-specific determinants such as the start, underwater phase, turn quality, and clean-swimming velocity, rather than by general swimming volume alone (Gao et al., 2025; Ruiz-Navarro et al., 2025).

Post-race cool-down is also widely used in practice, but its benefits are not uniform across recovery outcomes, which means such prescriptions should not be treated as generic add-ons (Van Hooren and Peake, 2018). General recovery literature indicates that active cool-down does not universally improve all markers of recovery or subsequent performance (Van Hooren and Peake, 2018). In swimming, however, several studies have shown that post-exercise recovery responses depend on how active recovery is prescribed, including its intensity, duration, and structure (McMaster et al., 1989; Greenwood et al., 2008; Vescovi et al., 2011; Sorgente et al., 2023). Research in swimmers has demonstrated that active recovery may influence blood lactate clearance and subsequent sprint performance, but inappropriate recovery intensity or poorly matched recovery structure may reduce rather than enhance recovery quality (McMaster et al., 1989; Greenwood et al., 2008). In addition, competition-based evidence suggests that blood lactate dynamics and recovery needs vary across swimmers and situations, supporting the idea that post-race relaxation or cool-down should remain feasible, individualized, and cautiously framed rather than presented as universally effective (Vescovi et al., 2011; Sorgente et al., 2023).

Therefore, evaluating AI-generated plans in this context should consider not only textual completeness and usability, but also whether the generated prescriptions are physiologically coherent for sprint-swimming preparation and recovery. Existing evaluation studies have mainly focused on general exercise prescription, running, resistance or hypertrophy training, and comparisons across ChatGPT versions, whereas swimming-specific evidence remains limited (Dergaa et al., 2024; Düking et al., 2024; Genç et al., 2025; Havers et al., 2025b; Puce et al., 2026; Yang et al., 2026b). The available swimming study suggests that AI may be acceptable for simpler programming tasks, but still requires human supervision in more complex and high-intensity contexts (Puce et al., 2026). A further methodological concern is the stability of AI-generated training plans. In the present study, generation-level stability refers to the degree to which repeated outputs produced by the same AI tool under an identical prompt and standardized generation conditions yield similar expert-rated content-quality scores. It therefore reflects consistency across repeated generations rather than the absolute scientific quality, safety, or practical suitability of the generated content. Unlike fixed templates or deterministic rule-based systems, LLMs generate text through probabilistic next-token prediction and decoding processes, meaning that repeated use of the same prompt may not always produce identical outputs (Holtzman et al., 2019; Achiam et al., 2023). This issue is practically important in sport and exercise prescription, where small differences in exercise type, intensity, volume, sequencing, recovery duration, or safety guidance may influence both feasibility and risk. Previous expert-evaluation studies have shown that AI-generated exercise plans are not uniformly optimal and that output quality depends partly on prompt detail and model choice (Dergaa et al., 2024; Düking et al., 2024; Genç et al., 2025; Havers et al., 2025b). More directly, Havers et al. examined the reproducibility of hypertrophy-related resistance training plans when the same prompts were provided multiple times, while Yang et al. used repeated prompt inputs and the coefficient of variation CV% to quantify the output variability of ChatGPT-generated training programs (Havers et al., 2025b; Yang et al., 2026b). Yang et al. further showed that even repeated inputs with the same information granularity could produce structurally and qualitatively different outputs, despite greater stability under detailed prompts (Yang et al., 2026b). Therefore, evaluating a single AI-generated plan may not sufficiently represent a model’s broader performance profile. In the present study, each AI model generated five independent outputs under the same standardized prompt, enabling assessment of both average expert-rated quality and within-model output stability in a sprint-swimming race-day scenario. Race-day warm-up and immediate post-race cool-down in sprint swimming are not merely organizational coaching routines; they require the translation of exercise physiology principles into practical decisions about activation, intensity, fatigue control, transition management, and recovery. To date, there is still a lack of comparative evidence on whether different LLMs generate meaningfully different competition-day warm-up and immediate post-race cool-down plans for sprint swimming under the same prompt conditions (Fradkin et al., 2010; Neiva et al., 2014a; McGowan et al., 2016; Van Hooren and Peake, 2018; Ramos-Campo et al., 2020; McKenzie et al., 2022; Puce et al., 2026). Because the 100-m freestyle is a sprint event in which pre-race preparation must optimize readiness without causing unnecessary fatigue, and post-race guidance must remain concise, feasible, and recovery-oriented, this scenario provides a demanding test of AI-generated content quality (Neiva et al., 2014a; McGowan et al., 2016; Van Hooren and Peake, 2018; Ramos-Campo et al., 2020; McKenzie et al., 2022). The present study therefore aimed to compare the expert-rated content quality and generation-level stability of race-day warm-up and immediate post-race cool-down plans generated by four publicly accessible LLM-based AI tools, and to clarify how these tools may support coach-supervised drafting, comparison, and refinement of swimming race-day preparation plans. Specifically, we examined: (1) whether overall expert-rated quality differed among the four AI models; (2) whether model differences varied between warm-up and cool-down sections; (3) how the models performed across predefined content-quality domains; and (4) whether repeated outputs generated under the same standardized prompt showed different levels of stability. We expected that expert-rated quality would differ across models and that repeated generations would show non-negligible variability even under the same prompt condition.

## 2 Methods

### 2.1 Study design

This study used a simulation-based content-analysis design with a within-rater repeated-measures structure. Four publicly accessible AI language models were compared: Web GPT-5.2 Thinking (OpenAI ChatGPT), Web Gemini 3 Flash Thinking (Google Gemini), Web DeepSeek-V3.2 (DeepSeek), and Web Doubao-Seed-2.0-pro (ByteDance Doubao). Each model generated five independent outputs using the same standardized detailed prompt. Each output contained two sections: a race-day warm-up plan and an immediate post-race cool-down plan for a simulated 100-m freestyle swimmer.

All outputs were generated in separate, context-free sessions to reduce carryover effects. The final experimental materials therefore included 20 AI-generated outputs, corresponding to 4 AI models × 5 independent generations, with each output including both warm-up and cool-down sections. Before expert evaluation, all plans were reformatted into a unified template, stripped of model-identifying information, assigned non-informative codes, and presented in randomized order. The same expert panel rated all plans independently and anonymously, allowing within-rater comparisons across AI models, generated versions, and plan sections.

### 2.2 AI models

Four publicly accessible, web-based large language model (LLM) tools were purposively selected: ChatGPT, Gemini, DeepSeek, and Doubao. The selection was based on three considerations. First, all four tools were directly accessible through general-purpose web interfaces and could realistically be used by coaches, athletes, and sport practitioners seeking rapid support for training-related content. Second, the tools were developed by independent providers and therefore represented different model and platform ecosystems rather than multiple versions from a single developer. Third, ChatGPT and Gemini represented internationally available artificial intelligence platforms, whereas DeepSeek and Doubao represented China-based platforms with practical relevance to Chinese-language and domestic sport settings. Together, this selection broadened the practical relevance of the comparison across provider, regional, and user contexts.

All models were accessed through their official web interfaces. The model names and version labels were recorded as displayed in the web interface at the time of data generation: Web GPT-5.2 Thinking (OpenAI ChatGPT), Web Gemini 3 Flash Thinking (Google Gemini), Web DeepSeek-V3.2 (DeepSeek), and Web Doubao-Seed-2.0-pro (ByteDance Doubao). No additional fine-tuning, external plugins, retrieval-augmented tools, uploaded reference files, or follow-up prompts were used during content generation. To improve reproducibility, the standardized prompts, model access information, and complete AI-generated outputs were retained and are provided in the **Supplementary Materials**.

### 2.3 Scenario and prompt design

A standardized, task-specific prompt was developed to elicit two race-day planning components for the same simulated athlete: a pre-race warm-up plan and an immediate post-race cool-down plan. The athlete was defined as a 16-year-old male swimmer with Chinese national Level-2 classification competing in the 100-m freestyle. This hypothetical profile was selected to provide a clearly defined sprint-swimming scenario requiring balanced physiological activation, technical preparation, and fatigue control.

The same athlete information and task requirements were submitted to all four tools. The standardized prompt was submitted in Chinese, and all generated outputs were retained and evaluated in Chinese. The prompt specified key contextual factors, including competition schedule, facilities, time constraints, and equipment, and required table-formatted outputs detailing activity type, duration, intensity, sequencing, and implementation notes.

**Figure 1:**
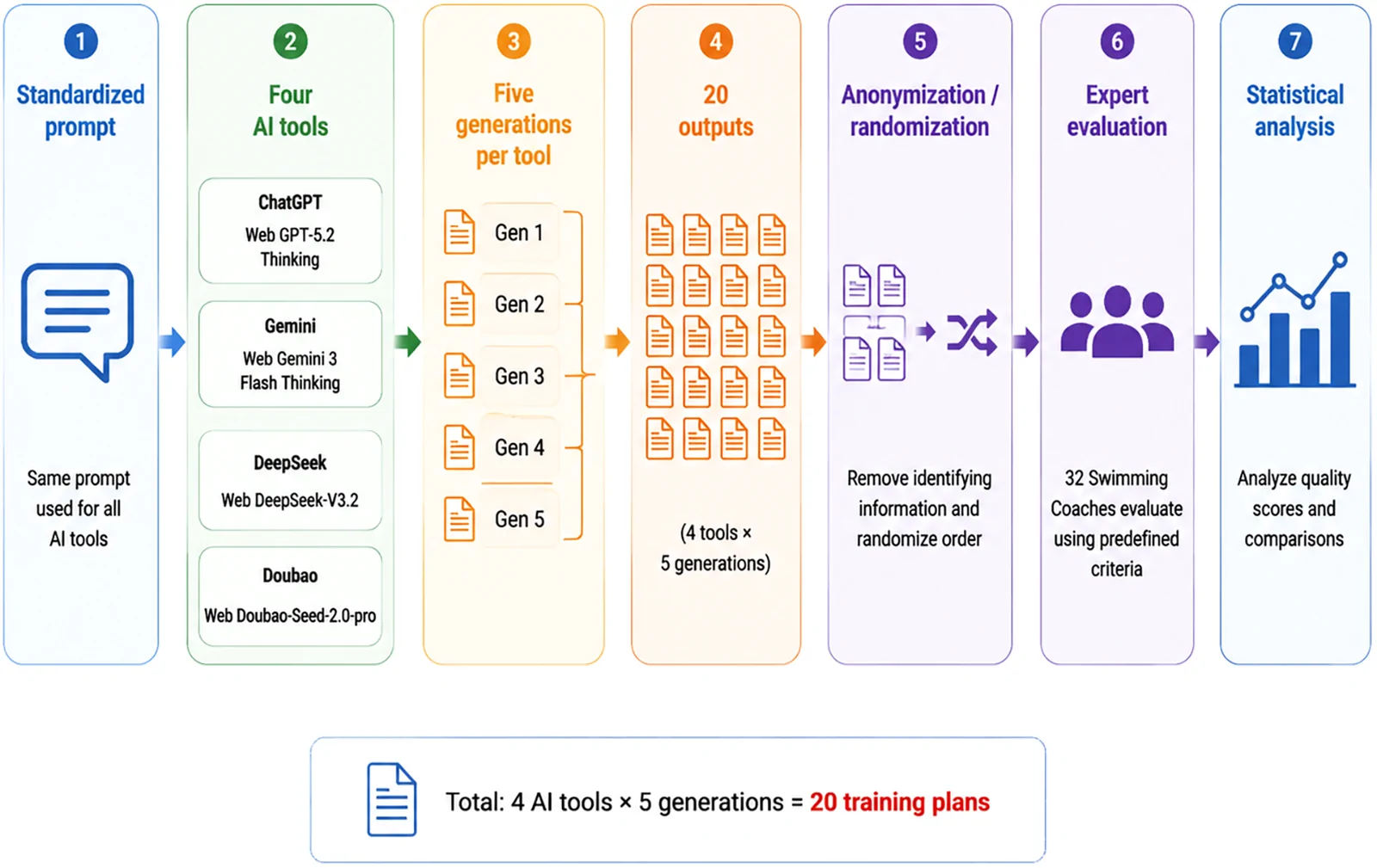
Study design for generating and evaluating AI-generated swimming race-day plans. Four AI models each generated five plans from a standardized prompt, yielding 20 plans that were evaluated by 32 swimming coaches using predefined quality criteria.

These specifications aimed to reduce ambiguity and support consistent expert evaluation across predefined quality domains. The study workflow is summarized in **Figure 1**, and the complete standardized prompt is provided in **Supplementary Note 1**.

### 2.4 Generation procedure and blinding

Each artificial intelligence tool generated five independent outputs from the same standardized prompt on 1 March 2026. All generations were completed by the same researcher using one newly created free account for each AI platform, with the same account used for all five generations within each platform. Conversation history, memory, and personalization functions were disabled, and no optional web-search function was manually enabled. Each generation was conducted in a new, context-free dialogue window. No follow-up prompts, corrective instructions, partial regeneration, or manual refinement were used, and the first complete response was retained. Each response included a race-day warm-up plan and an immediate post-race cool-down plan.

A single-turn procedure was used to standardize interaction conditions and compare the tools’ initial responses to identical input. The study therefore assessed first-response quality and stability under controlled conditions rather than the maximum quality achievable through iterative coach–artificial intelligence interaction.

The original outputs were archived and reformatted into a unified template without changing activities, sequence, duration, distance, repetitions, intensity, rest intervals, safety statements, or implementation notes. Model-identifying information was removed, and outputs were assigned non-informative codes from A1–1 to A4-5. The coded plans were presented in an order randomized using the RAND function in Microsoft Excel and rated independently and anonymously by experts blinded to the generating tool. The coding key was withheld until evaluation was completed.

### 2.5 Expert raters

A total of 32 experts participated in the evaluation. Experts were eligible if they had at least five years of experience in coaching or teaching swimming and had direct experience with 100-m sprint swimming instruction, training, or competition preparation. Experts were recruited from sport training, swimming training, physical education teaching, and sport rehabilitation backgrounds. Individuals were excluded if they had no experience with 100-m sprint swimming or if they were involved in generating, formatting, or anonymizing the AI outputs.

As shown in **Table 1**, the expert panel included raters with backgrounds in sport training (46.88%), swimming training (34.38%), physical education teaching (15.63%), and sport rehabilitation (3.13%). Regarding coaching settings, 50.00% worked with school teams, 25.00% with city teams, 15.63% with provincial teams, and 9.38% with national teams. Most experts had 5–9 years (46.88%) or ≥15 years (40.63%) of coaching experience. In terms of the highest athlete level coached, 50.00% had coached national level 1 athletes, 31.25% national level 2 athletes, 15.63% national elite athletes, and 3.13% international elite athletes. Ten experts (31.25%) reported prior experience using AI-generated training content. All expert characteristics were self-reported and are summarized in **Table 1**.

**Table 1:** Expert characteristics.

Characteristic	Category	n	%
Education level	PhD	2	6.25
	Bachelor	18	56.25
	Master	12	37.50
Major background	PE teaching	5	15.63
	Swimming training	11	34.38
	Sport rehabilitation	1	3.13
	Sport training	15	46.88
Coaching organization	National team	3	9.38
	City team	8	25.00
	School team	16	50.00
	Provincial team	5	15.63
Athlete level	National level 1	16	50.00
	National level 2	10	31.25
	National elite	5	15.63
	International elite	1	3.13
100m teaching experience	Yes	32	100.00
Coaching years	10-14	4	12.50
	≥15	13	40.63
	5-9	15	46.88
Used AI before	No	22	68.75
	Yes	10	31.25
Total		32	100.0

Data are presented as n (%), based on unique experts (N = 32). Education level, major background, coaching organization, highest athlete level coached, 100-m sprint coaching experience, coaching years, and prior use of AI-generated training plans were self-reported. Percentages may not sum to 100% due to rounding.

Ethical approval for this study was obtained from the Sports Medicine Ethics Committee of the School of Physical Education, Sichuan University (approval number: YDYXLL-2026-02). All expert raters provided written informed consent before participation. Consent forms were stored separately from rating data, and all ratings were collected anonymously, analyzed only in aggregate form, and reported without personally identifiable information.

### 2.6 Rating instrument

AI-generated training plans should be evaluated against practical criteria similar to those used for coach-designed plans. Previous coach-rating studies have shown that output quality varies across model versions and depends partly on the amount and structure of athlete- and context-specific information provided, supporting the use of a structured, domain-based rubric rather than a single overall score (Dergaa et al., 2024; Düking et al., 2024; Havers et al., 2025b).

The rubric was developed to assess 10 predefined content-quality domains aligned with the questionnaire for swimming race-day warm-up and post-race cool-down plans. Scientific validity assessed whether the plan followed basic exercise prescription principles, specified training dose clearly, and avoided claims beyond the evidence (Fradkin et al., 2010; Garber et al., 2011). Structural completeness assessed whether the plan had a complete and logically sequenced structure, moving from general preparation to event-specific readiness in the warm-up and from post-race exertion toward gradual recovery in the cool-down (Bishop, 2003a; Fradkin et al., 2010). Sport-specific targeting assessed whether the plan addressed sprint-swimming demands, including high-speed readiness, technical rhythm, and transition into race conditions (Ramos-Campo et al., 2020; McKenzie et al., 2022). Safety assessed whether the plan included appropriate cautions, avoided excessive or risky content, and reflected basic risk-screening considerations (Riebe et al., 2015). Individualization assessed whether the plan used the provided athlete profile, time constraints, equipment, and competition schedule rather than offering generic advice (Düking et al., 2024; Havers et al., 2025a). Practical applicability assessed whether the plan could be executed as written, with clear sets, distances, time blocks, rest intervals, and usable intensity guidance (Garber et al., 2011).

Rationality of arrangement assessed whether the warm-up enhanced readiness without unnecessary fatigue and whether the cool-down promoted gradual downregulation without overstating recovery effects (Bishop, 2003a; Van Hooren and Peake, 2018). Clarity and dose specification assessed whether the prescription was clear, consistent, and sufficiently detailed, including practical intensity anchors such as rating of perceived exertion (RPE) (Borg, 1982). Innovation assessed whether any novel elements were feasible, optional, and safe, particularly for re-warm-up or potentiation strategies in sprint swimming (Abbes et al., 2018; Ramos-Campo et al., 2020; McKenzie et al., 2022).

Overall quality captured the expert’s summary judgment of whether the plan would be acceptable for use in the given setting (Düking et al., 2024; Havers et al., 2025a).

The instrument contained 40 item-level ratings, with 20 items assessing the race-day warm-up section and 20 items assessing the immediate post-race cool-down section. Each item was rated on a 1–5 Likert scale, with higher scores indicating better content quality. To improve rating consistency, each domain included an operational definition and item-specific scoring anchors. The full rating instrument is provided in **Supplementary Table 1**. For the domain-level analyses, ratings for all items assigned to the same domain were averaged across the warm-up and cool-down sections and across the five repeated generations at the rater level, yielding one domain score per rater for each AI model and domain.

The rating instrument was developed through a literature-informed and expert-refined process. First, the research team identified candidate domains and items from previous studies evaluating AI-generated exercise or training plans and from swimming-specific literature on race-day warm-up, transition strategies, sprint-swimming demands, and post-exercise recovery. Second, the item pool was adapted to the practical characteristics of a 100-m freestyle race-day scenario, with particular attention to event-specific readiness, transition feasibility, dose clarity, safety, and immediate post-race recovery. Third, three swimming-training experts reviewed the initial item pool and provided structured feedback on item relevance, wording clarity, domain alignment, scoring feasibility, and practical applicability. Based on their feedback, ambiguous wording was revised, overlapping expressions were reduced, and several items were refined to better distinguish warm-up and cool-down requirements.

Content validity was therefore established qualitatively through literature-based item development, expert review, and pilot testing rather than through a quantified content validity index. Because the instrument was developed specifically for this study, its scores should be interpreted as expert-rated content-quality indicators within the present swimming race-day scenario rather than as a fully validated general-purpose scale.

### 2.7 Pilot testing and rater training

Before formal data collection, the rating instrument was pilot-tested by ten experts who were not included in the final expert-rating panel. The pilot test examined item clarity, scoring feasibility, and the interpretability of the rating anchors. Feedback from the pilot test was used to refine wording, reduce ambiguity, and improve consistency between item definitions and the 1–5 scoring anchors.

Before the formal evaluation, all expert raters received standardized rating instructions, operational definitions for each item, and anchored scoring examples. Raters were allowed to ask procedural questions before scoring, but no discussion of specific AI-generated plans was permitted during the formal evaluation. This procedure was intended to improve scoring consistency while preserving independent expert judgment.

### 2.8 Sample size justification

An *a priori* sample size estimation was performed using G*Power software (version 3.1.9.7), following established power-analysis procedures described by Faul et al. (2009) and conventional effect-size guidance proposed by Cohen (Faul et al., 2009; Cohen, 2013). Because the same panel of experts evaluated outputs generated by all four large language models (LLMs), the study was treated as a repeated-measures design with four measurements. For the *a priori* calculation, a medium effect size was assumed (f = 0.25), with an alpha level of 0.05, statistical power of 0.80, one group, four repeated measurements, a correlation among repeated measures of 0.50, and a nonsphericity correction of 1.0. Under these assumptions, the minimum required sample size was 24 expert raters, with an actual power of 0.817. The final sample included 32 experts, exceeding the estimated minimum requirement. This sample size was also considered pragmatically appropriate in light of comparable expert-evaluation studies on AI-generated training or exercise plans, which have used panels of 7, 10, and 12 expert raters, while a swimming-specific AI study involved 23 coaches in evaluating AI-generated training content (Düking et al., 2024; Genç et al., 2025; Havers et al., 2025b; Puce et al., 2026). This calculation was used as a pragmatic reference for the repeated-measures comparison of the four AI models; the primary item-level analysis was conducted using a cumulative link mixed model (CLMM), which further accounted for repeated ratings by experts, item-level clustering, and generation-level variation.

### 2.9 Statistical analysis

Before analysis, the dataset was screened for completeness, coding consistency, and valid rating ranges. Because each expert rated multiple items across AI models, generated versions, and plan sections, ratings were retained at the item level for the primary analysis. Descriptive statistics were calculated for item-level ratings and aggregated warm-up, cool-down, and overall mean scores, and are reported as mean ± standard deviation (SD).

Inter-rater reliability was assessed before the main comparisons. Intraclass correlation coefficients (ICCs) were calculated for aggregated warm-up, cool-down, and overall scores using a two-way random-effects, absolute-agreement model. Both single-measure and average-measure ICCs were reported. Krippendorff’s alpha with an ordinal metric was used for item-level Likert ratings. Detailed results are provided in **Supplementary Tables 2**, **3**.

The primary analysis used a cumulative link mixed model (CLMM), as the outcome was an ordinal 1–5 Likert rating with repeated measures. The model included fixed effects for AI model, section, and their interaction, with random intercepts for rater, item, and generation ID:rating ~ model * section + (1 | rater) + (1 | item) + (1 | generation_id).

When significant model or interaction effects were found, section-specific *post hoc* comparisons were conducted using estimated marginal means with Holm adjustment. CLMM estimates were interpreted as log-odds differences, with positive values indicating higher odds of higher ratings.

Exploratory domain-level analyses compared AI models across the 10 predefined content-quality domains. Ratings were aggregated within each domain at the rater level, analyzed using Friedman tests, corrected using Holm adjustment, and reported with Kendall’s W as the effect size.

Generation-level stability was assessed across the five outputs generated by each model using the mean, minimum, maximum, range, SD, and coefficient of variation. The coefficient of variation was calculated as SD/mean × 100. Lower variability indicated greater output stability, but not necessarily higher content quality. Because each model generated five outputs, stability findings were interpreted as descriptive and exploratory. All analyses were conducted in R. Statistical significance was set at p < 0.05. Findings should be interpreted as evidence of expert-perceived content quality and output stability, not direct evidence of training effectiveness, physiological readiness, recovery benefit, or athlete adherence.

## 3 Results

### 3.1 Descriptive statistics of expert ratings

Descriptive statistics were calculated to summarize expert-rated warm-up, cool-down, and overall quality scores for each AI model after averaging item ratings within each section for each expert and generated version.

**Table 2** presents the descriptive statistics of expert ratings for the AI-generated warm-up, cool-down, and overall plans. Each AI model contributed 160 expert-by-generation records, corresponding to 32 experts evaluating five independently generated versions. ChatGPT had the highest descriptive overall mean score (4.23 ± 0.22), with similar ratings for the warm-up (4.17 ± 0.24) and cool-down (4.29 ± 0.25) sections. Gemini and DeepSeek showed closely comparable overall mean scores (3.94 ± 0.22 and 3.93 ± 0.26, respectively). Their section-specific patterns differed slightly: Gemini had a somewhat higher warm-up score (3.97 ± 0.20) than cool-down score (3.91 ± 0.30), whereas DeepSeek had similar warm-up (3.92 ± 0.28) and cool-down (3.94 ± 0.30) scores. Doubao had the lowest descriptive scores across all outcomes, with a warm-up score of 3.13 ± 0.24, a cool-down score of 3.31 ± 0.30, and an overall score of 3.22 ± 0.26.

**Table 2:** Descriptive statistics of expert ratings for AI-generated warm-up and cool-down plans.

AI model	n	Warm-up score Mean ± SD	Cool-down score Mean ± SD	Overall score Mean ± SD
ChatGPT	160	4.17 ± 0.24	4.29 ± 0.25	4.23 ± 0.22
Gemini	160	3.97 ± 0.20	3.91 ± 0.30	3.94 ± 0.22
DeepSeek	160	3.92 ± 0.28	3.94 ± 0.30	3.93 ± 0.26
Doubao	160	3.13 ± 0.24	3.31 ± 0.30	3.22 ± 0.26

Scores are section-level mean ratings on a 1–5 Likert scale after averaging item ratings within each section for each expert and generated version. Warm-up scores were calculated from 20 warm-up items, cool-down scores from 20 cool-down items, and overall scores from all 40 items. n denotes the number of expert-by-generation records for each model (32 experts × 5 generated versions). SD, standard deviation.

These descriptive results indicated visible differences in expert-rated content quality across AI models. Inferential analysis was then conducted using item-level ordinal ratings to account for repeated observations nested within raters, items, and generated versions.

Inter-rater reliability supported the use of aggregated expert ratings for subsequent analyses. For aggregated scores, single-measure ICCs were 0.801 for warm-up, 0.660 for cool-down, and 0.770 for overall scores, while average-measure ICCs were 0.992, 0.984, and 0.991, respectively. Krippendorff’s alpha values for item-level ordinal ratings were 0.559 for warm-up, 0.536 for cool-down, and 0.548 overall, indicating moderate single-item agreement. These results suggest high reliability for aggregated mean ratings, although some rater variability remained at the item level. Detailed results are provided in **Supplementary Tables 2**, **3**.

### 3.2 Primary CLMM results

A cumulative link mixed model was used as the primary analysis to test model differences in item-level ratings and to examine whether these differences varied between the warm-up and cool-down sections.

**Table 3** presents the CLMM results for AI model differences by section. The likelihood-ratio test showed a significant model × section interaction, χ²(3) = 136.23, p <.001, indicating that the pattern of model differences varied between the warm-up and cool-down sections. In the warm-up section, ChatGPT had higher odds of receiving higher ratings than DeepSeek (estimate = 0.823, SE = 0.235, z = 3.509, p = .001) and Doubao (estimate = 2.933, SE = 0.235, z = 12.462, p <.001). The ChatGPT–Gemini contrast followed the same direction but reached only the conventional significance threshold after adjustment (estimate = 0.527, SE = 0.235, z = 2.241, p = .050), so this result was interpreted cautiously. Gemini and DeepSeek did not differ significantly in the warm-up section (p = .206), whereas both were rated higher than Doubao (both p <.001).

**Table 3:** Cumulative link mixed model results for AI model differences by section.

Analysis	Contrast	Estimate	SE	Test statistic	p value
Panel A. Model x section interaction					
Likelihood-ratio test	Model x section	—	—	χ²(3) = 136.23	<.001
Panel B. Pairwise model comparisons within warm-up section					
Warm-up	ChatGPT - Gemini	0.527	0.235	z = 2.241	.050
Warm-up	ChatGPT - DeepSeek	0.823	0.235	z = 3.509	.001
Warm-up	ChatGPT - Doubao	2.933	0.235	z = 12.462	<.001
Warm-up	Gemini - DeepSeek	0.297	0.235	z = 1.263	.206
Warm-up	Gemini - Doubao	2.406	0.235	z = 10.227	<.001
Warm-up	DeepSeek - Doubao	2.110	0.235	z = 8.986	<.001
Panel C. Pairwise model comparisons within cool-down section					
Cool-down	ChatGPT - Gemini	1.203	0.235	z = 5.124	<.001
Cool-down	ChatGPT - DeepSeek	1.108	0.235	z = 4.719	<.001
Cool-down	ChatGPT - Doubao	2.872	0.235	z = 12.206	<.001
Cool-down	Gemini - DeepSeek	-0.095	0.234	z = -0.408	.684
Cool-down	Gemini - Doubao	1.669	0.235	z = 7.115	<.001
Cool-down	DeepSeek - Doubao	1.764	0.234	z = 7.523	<.001
Panel D. Within-model section comparisons					
ChatGPT	Warm-up - cool-down	-0.387	0.326	z = -1.188	.235
Gemini	Warm-up - cool-down	0.290	0.326	z = 0.889	.374
DeepSeek	Warm-up - cool-down	-0.102	0.326	z = -0.314	.753
Doubao	Warm-up - cool-down	-0.448	0.326	z = -1.377	.169

CLMM, cumulative link mixed model; SE, standard error. The dependent variable was the ordinal 1–5 Likert rating. The model included fixed effects for AI model, section, and their interaction, with random intercepts for rater, item, and generation ID. Estimates are log-odds differences from the cumulative link mixed model. A positive estimate indicates that the first model or section in the contrast had higher odds of receiving higher ratings than the comparison group. Pairwise comparisons were adjusted using the Holm method. The p value for the model x section interaction was obtained from the likelihood-ratio test comparing the main-effects model with the interaction model.

In the cool-down section, ChatGPT had higher odds of receiving higher ratings than Gemini (estimate = 1.203, SE = 0.235, z = 5.124, p <.001), DeepSeek (estimate = 1.108, SE = 0.235, z = 4.719, p <.001), and Doubao (estimate = 2.872, SE = 0.235, z = 12.206, p <.001). Gemini and DeepSeek again did not differ significantly from each other (p = .684), but both were rated higher than Doubao (both p <.001). Within-model section comparisons showed no significant warm-up–cool-down differences for ChatGPT (p = .235), Gemini (p = .374), DeepSeek (p = .753), or Doubao (p = .169).

After accounting for rater-, item-, and generation-level clustering, the primary CLMM results indicated a section-dependent model effect, with ChatGPT tending to receive higher ratings, Gemini and DeepSeek showing broadly comparable intermediate performance, and Doubao receiving lower ratings in both sections.

### 3.3 Exploratory domain-level analysis

Exploratory domain-level analyses were conducted to describe whether the model differences observed in the primary analysis were concentrated in specific content-quality domains.

**Figure 2:**
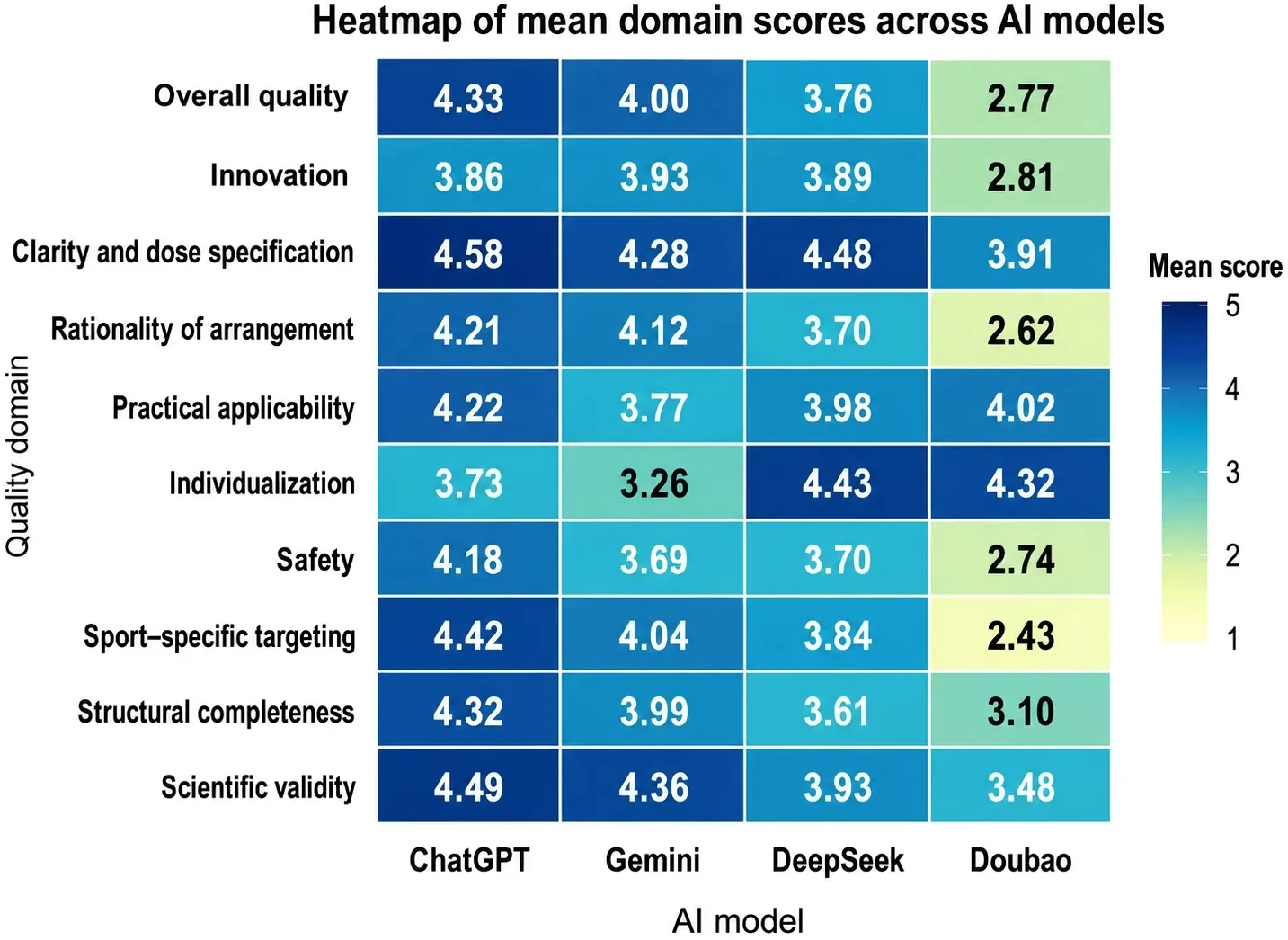
Descriptive heatmap of mean domain-level scores across AI models. Cell values represent mean domain scores averaged across raters and repeated generations. Darker shading indicates higher scores on the 1–5 rating scale. The heatmap is intended to facilitate visual comparison of domain-level rating patterns across models. For inferential results, see **Table 4**, where domain-level scores are summarized as median [interquartile range] and compared using Friedman tests with Holm correction.

**Table 4** presents the domain-level comparisons, and **Figure 2** shows the heatmap of mean domain scores across AI models. Friedman tests showed significant differences among the four AI models across all 10 domains after Holm correction (all adjusted p <.001). The effect sizes varied across domains, with Kendall’s W ranging from 0.334 for practical applicability to 0.927 for structural completeness. This indicated that domain-level model differences were more pronounced for some quality dimensions than for others.

**Table 4:** Exploratory domain-level analysis of expert ratings across AI models.

Domain	ChatGPT	Gemini	DeepSeek	Doubao	Friedman χ²	df	Adjusted p	Kendall’s W
Scientific validity	4.50 [4.45, 4.55]	4.40 [4.25, 4.50]	4.00 [3.80, 4.06]	3.50 [3.45, 3.70]	79.89	3	<0.001	0.832
Structural completeness	4.35 [4.25, 4.40]	4.03 [3.84, 4.15]	3.62 [3.40, 3.80]	3.10 [2.94, 3.25]	88.95	3	<0.001	0.927
Sport-specific targeting	4.43 [4.35, 4.50]	4.03 [3.89, 4.20]	3.88 [3.75, 4.10]	2.50 [2.25, 2.75]	81.91	3	<0.001	0.853
Safety	4.15 [4.10, 4.30]	3.70 [3.50, 3.91]	3.80 [3.60, 3.90]	2.95 [2.45, 3.05]	82.52	3	<0.001	0.860
Individualization	3.75 [3.65, 3.80]	3.23 [3.00, 3.45]	4.50 [4.25, 4.56]	4.38 [4.29, 4.51]	76.53	3	<0.001	0.797
Practical applicability	4.22 [4.14, 4.30]	3.85 [3.70, 4.00]	4.03 [3.89, 4.16]	4.00 [3.90, 4.25]	32.03	3	<0.001	0.334
Rationality of arrangement	4.20 [4.15, 4.26]	4.05 [3.89, 4.35]	3.70 [3.56, 3.86]	2.60 [2.44, 2.90]	76.92	3	<0.001	0.801
Clarity and dose specification	4.60 [4.49, 4.65]	4.25 [4.00, 4.50]	4.43 [4.27, 4.76]	4.00 [3.71, 4.06]	42.44	3	<0.001	0.442
Innovation	3.85 [3.79, 4.00]	4.00 [3.90, 4.00]	4.00 [3.75, 4.06]	2.75 [2.50, 3.00]	61.48	3	<0.001	0.640
Overall quality	4.35 [4.25, 4.40]	4.00 [3.84, 4.18]	3.88 [3.65, 3.91]	2.95 [2.49, 3.00]	76.93	3	<0.001	0.801

Values are presented as median [interquartile range]. Domain-level scores were calculated by averaging item ratings across the warm-up and cool-down sections and across five repeated generations at the rater level. Friedman tests were used to compare the four AI models within each domain. Adjusted p values were obtained using the Holm correction. Kendall’s W is reported as the effect size. AI, artificial intelligence; IQR, interquartile range.

Descriptively, ChatGPT showed higher domain-level scores in several core quality domains, including scientific validity, structural completeness, sport-specific targeting, safety, rationality of arrangement, clarity and dose specification, and overall quality. Gemini and DeepSeek generally showed intermediate profiles across most domains. DeepSeek showed the highest median score for individualization, while Doubao also performed relatively well in this domain. Doubao showed lower scores in several sport-specific and structural domains, including sport-specific targeting, safety, rationality of arrangement, innovation, and overall quality; however, its scores for practical applicability and individualization were not as low as its scores in other domains.

These exploratory domain-level results suggest that model differences were not attributable to a single rating domain. Instead, the four AI tools showed distinct domain-specific profiles. Because *post hoc* pairwise comparisons were not conducted for every domain, these domain-specific patterns should be interpreted descriptively. This decision was made because the domain-level analyses were exploratory and the primary inferential model comparisons were already addressed by the CLMM, thereby limiting additional multiple testing.

### 3.4 Generation-level stability analysis

An exploratory generation-level stability analysis was conducted to describe how much expert-rated quality varied across the five independently generated versions within each AI model.

**Figure 3:**
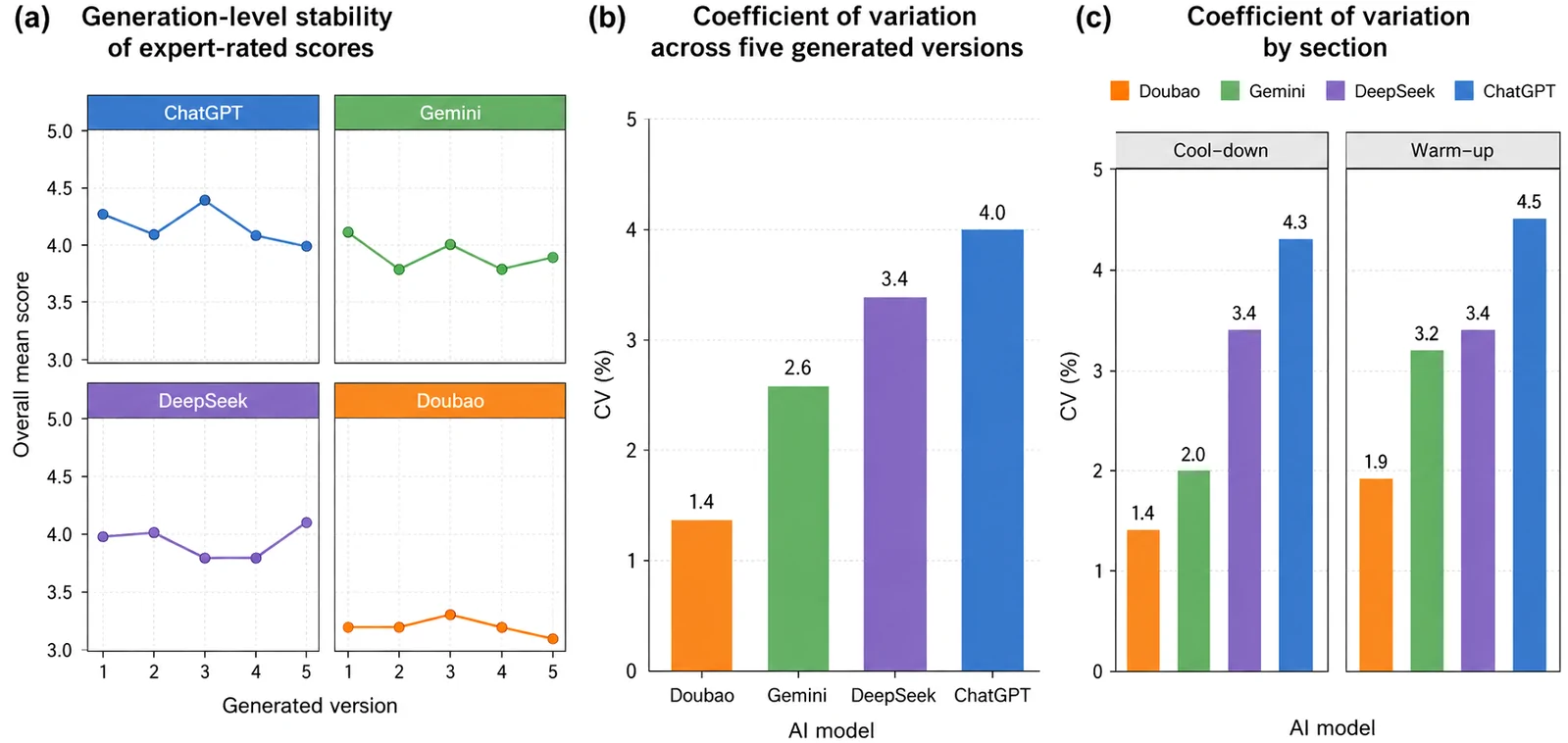
Generation-level stability of expert-rated scores across five independently generated versions. **(a)** Overall mean scores across the five generated versions for each AI model. **(b)** Coefficient of variation (CV%) for overall quality scores across the five generated versions. **(c)** Section-specific CV% for warm-up and cool-down scores. Lower CV% indicates lower repeated-generation variability.

**Table 5** presents the generation-level stability results, and **Figure 3** visualize the overall and section-specific variability patterns. For overall quality scores, Doubao had the lowest variability across the five generated versions (range = 0.134, SD = 0.049, CV = 1.525%), followed by Gemini (range = 0.245, SD = 0.101, CV = 2.561%) and DeepSeek (range = 0.305, SD = 0.130, CV = 3.295%). ChatGPT had the highest variability (range = 0.434, SD = 0.169, CV = 3.989%). However, this variability pattern did not match the mean quality pattern: ChatGPT had the highest overall mean score (4.233), whereas Doubao had the lowest overall mean score (3.222).

**Table 5:** Generation-level stability of expert-rated quality scores across five independently generated versions.

Section	AI model	n	Mean	Minimum	Maximum	Range	SD	CV (%)
Overall	ChatGPT	5	4.233	4.030	4.464	0.434	0.169	3.989
Overall	Gemini	5	3.943	3.828	4.073	0.245	0.101	2.561
Overall	DeepSeek	5	3.932	3.784	4.090	0.305	0.130	3.295
Overall	Doubao	5	3.222	3.149	3.284	0.134	0.049	1.525
Warm-up	ChatGPT	5	4.173	3.953	4.456	0.503	0.184	4.407
Warm-up	Gemini	5	3.974	3.830	4.127	0.297	0.127	3.201
Warm-up	DeepSeek	5	3.918	3.752	4.073	0.322	0.130	3.324
Warm-up	Doubao	5	3.131	3.030	3.186	0.156	0.060	1.926
Cool-down	ChatGPT	5	4.293	4.106	4.494	0.388	0.183	4.269
Cool-down	Gemini	5	3.912	3.827	4.020	0.194	0.077	1.973
Cool-down	DeepSeek	5	3.945	3.800	4.106	0.306	0.132	3.342
Cool-down	Doubao	5	3.313	3.269	3.381	0.113	0.045	1.354

Values were calculated at the generation level after averaging expert ratings within each generated version. n = number of independently generated versions. Range = maximum minus minimum generation-level mean score. CV (%) = SD/mean × 100. Lower SD and CV values indicate lower generation-level variability, but do not necessarily indicate higher content quality.

**Figure 3a** presents the generation-level line plot of overall quality scores across the five versions. The plot showed greater fluctuation for ChatGPT, whereas Doubao showed a flatter but consistently lower trajectory. **Figure 3b** further shows the overall coefficient of variation, confirming that Doubao had the lowest CV% and ChatGPT had the highest CV%. The section-specific coefficient of variation in **Figure 3c** showed a similar pattern: Doubao had the lowest CV% in both warm-up (1.926%) and cool-down (1.354%), whereas ChatGPT had the highest CV% in both warm-up (4.407%) and cool-down (4.269%).

These exploratory stability results indicate that generation-level consistency and expert-rated content quality did not necessarily move in the same direction. In this study, the most stable model was not the highest-rated model. Because each model generated only five repeated outputs, these findings should be interpreted as descriptive evidence of repeated-generation variability rather than definitive estimates of model reproducibility.

## 4 Discussion

### 4.1 Principal findings

The main finding of this study was that publicly accessible LLM-based AI tools could generate recognizable and generally structured race-day warm-up and immediate post-race cool-down plans, but differed in their ability to translate sprint-swimming physiological principles into coherent, safe, and practically usable prescriptions. Across the four tools, the generated plans commonly included a land-based component, a water-based component, intensity descriptors such as RPE, technical or race-related preparation, and post-race low-intensity recovery elements. Most outputs also attempted to organize the plan into time blocks, distances, exercise components, and practical reminders. This indicates that current AI tools have reached a level at which they can rapidly transform a standardized race-day scenario into an editable coaching draft.

However, the shared ability to produce structured plans did not mean that the four tools generated plans of equivalent quality. The model differences became clearer when the plans were evaluated against swimming-specific and expert-defined criteria. ChatGPT showed the most favorable expert-rated profile, with higher scores across several core domains, including scientific validity, structural completeness, sport-specific targeting, safety, rationality of arrangement, clarity and dose specification, and overall quality. Gemini and DeepSeek showed broadly comparable intermediate performance, whereas Doubao received lower ratings in several sport-specific, structural, safety-related, and overall quality domains. The CLMM results further indicated that model differences varied between the warm-up and cool-down sections, suggesting that AI performance should be interpreted separately for different plan components. In addition, generation-level stability did not correspond directly to content quality: Doubao was the most stable model but produced lower-rated outputs, whereas ChatGPT received the highest ratings but showed greater variability across repeated generations. Overall, these findings suggest that AI-generated swimming race-day plans should be evaluated from four perspectives: basic structural adequacy, swimming-specific relevance, section-specific performance, and repeated-generation stability.

### 4.2 Interpretation of model differences

The commonalities across the four AI tools may be explained partly by the standardized and detailed prompt used in this study. The prompt specified the swimmer profile, event, schedule, setting, equipment, and required table format, thereby giving all tools enough information to produce a basic warm-up and cool-down structure. This supports previous findings that more detailed input can improve the structure and apparent quality of AI-generated training plans (Dergaa et al., 2024; Düking et al., 2024; Havers et al., 2025b). In practical terms, this means that AI can already support coaches in routine planning tasks, especially when the task involves organizing known training components into a readable table, clarifying time allocation, and generating an initial plan that can be reviewed and edited.

The differences among models appeared to arise from how each tool prioritized different aspects of the same task. Under the tested prompt and model conditions, ChatGPT tended to provide a more balanced plan structure, clearer dose variables, and stronger links between the race-day goal and the warm-up or cool-down components. In the supplementary comparison of generated content, ChatGPT commonly maintained a moderate warm-up volume and included more race-relevant elements than the lower-rated model, such as start preparation, turn-related content, pre-race reminders, and psychological cues. This may explain why experts rated it higher in structural completeness, dose clarity, sport-specific targeting, and overall quality.

Gemini and DeepSeek showed intermediate but slightly different profiles. Gemini often provided rich pre-race reminders, psychological suggestions, and a relatively complete race-day communication structure. However, some Gemini outputs prescribed longer warm-up duration or larger water volume, which may require coach screening in a sprint race-day context where unnecessary fatigue should be avoided. DeepSeek showed relatively strong attention to individual adjustment, environmental adaptation, and safety-related reminders, but some outputs showed wider variation in intensity range, recovery volume, or cool-down structure. These features may explain why Gemini and DeepSeek received similar overall ratings in the present scenario while showing different practical strengths.

Doubao’s profile was different. Its outputs were highly consistent across repeated generations and often followed a stable time structure, but this stability was accompanied by reduced sport-specific detail. In the content comparison, Doubao frequently omitted check-in timing, pre-race reminders, psychological preparation, turn-related content, and start-specific preparation. This pattern suggests that a stable AI output may still be limited if it repeatedly reproduces a generic template. For 100-m freestyle race-day preparation, the key issue is not only whether a plan has a clear format, but whether it operationalizes the performance-relevant details that coaches consider important. A recent expert evaluation in sports injury care also found version-related differences between contemporary large language models under standardized prompt conditions, while emphasizing that expert-rated performance does not establish autonomous decision-making capability and may change as models and web interfaces are updated (Kaya et al., 2026). These interpretations are specific to the model versions, web interfaces, athlete profile, and standardized prompt evaluated in this study and should not be regarded as permanent characteristics of the four tools.

### 4.3 Swimming-specific implications

The present findings should be interpreted within the specific logic of competitive swimming rather than general exercise prescription. A race-day plan for a 100-m freestyle swimmer is not simply a short preparatory or recovery routine, because sprint swimming performance depends on physiological readiness, technical execution, and phase-specific efficiency, including the start, underwater phase, turn, clean-swimming velocity, and finish (Marinho et al., 2020; Fernandes et al., 2024; Gao et al., 2025; Ruiz-Navarro et al., 2025). Therefore, an AI-generated plan may appear complete in format, but still be limited if it does not reflect the event-specific demands of sprint swimming.

For the pre-race warm-up, the central issue is whether the sequence and dose of each component can enhance readiness without inducing unnecessary fatigue. Warm-up should increase body and muscle temperature, enhance neuromuscular activation, support race-pace sensation, and maintain psychological preparedness (Bishop, 2003a; Bishop, 2003b; McGowan et al., 2015). In the 100-m freestyle context, these aims need to be translated into sprint-specific preparation, including explosive activation, start and turn readiness, underwater preparation, race-pace rehearsal, and management of the transition period between pool warm-up and race start (Neiva et al., 2014a, Neiva et al., 2014b; Neiva et al., 2017a; Neiva et al., 2017b; Abbes et al., 2018; Blazevich and Babault, 2019; Cuenca-Fernández et al., 2022; McKenzie et al., 2022).

The immediate post-race cool-down requires a different logic. It should support a feasible transition from maximal race effort toward low-intensity recovery, rather than adding further training stimulus. Although active cool-down is widely used, its effects vary according to intensity, duration, recovery outcome, and competitive context (McMaster et al., 1989; Greenwood et al., 2008; Toubekis et al., 2008; Vescovi et al., 2011; Van Hooren and Peake, 2018; Sorgente et al., 2023). More broadly, recovery should be considered in relation to accumulated load, fatigue, and the swimmer’s recovery status (Barbosa et al., 2010; Halson, 2014). These findings suggest that AI tools may help draft structured swimming plans, but their outputs must be reviewed by coaches or sport-science professionals, especially when intensity control, race-specific preparation, and recovery management are involved (Puce et al., 2026).

### 4.4 Stability versus quality

Generation-level stability and expert-rated quality should be treated as distinct dimensions of AI-generated training content. In this study, Doubao showed the lowest generation-level variability but received consistently lower ratings; conversely, ChatGPT received the highest expert ratings but showed greater variability across repeated generations. This suggests that stability does not necessarily indicate high quality, and higher-rated outputs may still fluctuate under the identical prompt conditions. For swimming race-day preparation, this distinction is important because small differences in exercise sequence, intensity, volume, rest intervals, or recovery guidance may affect feasibility and safety. These findings are consistent with previous studies showing that prompt detail, model choice, and repeated prompting can influence the quality and consistency of AI-generated training plans (Genç et al., 2025; Havers et al., 2025b; Yang et al., 2026b), and that fluency or structure should not be equated with accuracy, safety, or practical usability (Wilhelm et al., 2023; Zheng et al., 2025; Yang et al., 2026a). Therefore, coaches should avoid relying on a single AI-generated plan and should instead generate multiple outputs, compare them against swimming-specific criteria, and revise them through expert judgment.

### 4.5 Practical implications for AI-assisted swimming race-day preparation

The practical value of AI-generated race-day plans lies in their use within a coach-led human–AI interaction process, rather than in replacing coaching expertise. In swimming practice, coaches need plans that translate event-specific demands, race schedules, facility constraints, and athlete status into actionable decisions. Therefore, AI tools may be most useful as drafting and comparison aids that help organize plan structure, clarify dose variables, and identify possible plan components.

For 100-m freestyle race-day preparation, coaches may first provide a detailed prompt including the swimmer’s event, level, race schedule, warm-up pool conditions, available equipment, previous warm-up habits, and safety constraints. They may then generate several candidate plans and compare them against swimming-specific criteria. For the warm-up section, coaches should check whether the plan supports progressive activation, start and turn readiness, underwater preparation, race-pace rehearsal, transition-period management, and fatigue control. For the cool-down section, they should check whether the plan emphasizes low-intensity recovery, relaxation, feasibility, and consistency with the post-race schedule.

The choice of AI tool should depend on the coach’s practical aim. In the present scenario, ChatGPT appears most suitable when coaches need a relatively complete and higher-quality draft that integrates structure, dose clarity, safety, and sport-specific relevance. Because its repeated outputs were more variable, coaches should generate several versions and select the best elements. Gemini may be useful when coaches need richer pre-race communication, psychological reminders, and a detailed plan format, but its warm-up duration and total volume should be screened to avoid excessive loading. DeepSeek may be useful when coaches want additional individualization, environmental adaptation, and safety-related reminders, but its intensity ranges and recovery structure should be checked carefully. Doubao may be useful for quickly producing stable and concise templates, but its outputs require substantial coach revision before use in sprint-swimming race-day preparation because several key competition-specific elements may be missing.

Therefore, AI should be understood as a tool for improving coaching efficiency, not as an autonomous source of final race-day prescriptions. It can help coaches generate drafts, compare alternatives, specify timing and intensity, and prepare athlete-facing instructions. Final decisions on intensity, volume, sequencing, recovery, and individual adjustment should remain under coach supervision. This coach-led approach may allow AI tools to serve swimming training more practically while reducing the risk of generic or inappropriate prescriptions.

Recent reviews in clinical exercise physiology similarly conclude that generative artificial intelligence may support exercise-program design and professional efficiency, but should remain within professionally supervised workflows because evidence supporting its safe and autonomous implementation remains limited (Lederman et al., 2025).

Beyond these practical considerations, the use of LLM-generated race-day plans also raises ethical concerns related to responsibility, privacy, and potential model bias. Because fluent and well-structured outputs may encourage over-reliance, final race-day decisions should remain under qualified coach supervision. In real-world applications, coaches should also exercise caution when entering identifiable athlete information and recognize that differences among models may partly reflect variations in training-data coverage, alignment procedures, language, and cultural representation. Because all prompts and expert evaluations in the present study were conducted in Chinese, differences in Chinese-language coverage, swimming-specific terminology, and the representation of coaching conventions may also have influenced model performance, although these effects were not directly tested. As the present study examined only one 16-year-old male swimmer within a Chinese competitive classification context, potential demographic, linguistic, and cultural biases could not be assessed and should be addressed in future research.

## 5 Strengths and limitations

This study has several strengths. First, all AI-generated plans were produced using the same standardized detailed prompt, which reduced input-related differences and enabled a controlled comparison across models. Second, the study compared four publicly accessible AI tools, including both internationally prominent and China-based models, improving the practical relevance of the findings. Third, each model generated five independent outputs, allowing assessment of both expert-rated quality and repeated-generation stability rather than relying on a single output. Fourth, the plans were evaluated by 32 experts with experience in 100-m sprint swimming, using a 40-item multidimensional rating instrument. Finally, the use of cumulative link mixed models was appropriate for Likert-type ordinal ratings and accounted for the repeated-measures structure of raters, items, and generated outputs.

Several limitations should be noted. First, the use of a single simulated athlete profile limits generalizability to swimmers of other ages, sexes, competitive levels, health conditions, strokes, race distances, and competition schedules. In addition, because the simulated profile represented only a 16-year-old male swimmer within a Chinese competitive classification context, the study was not designed to determine whether model performance differs systematically across sex, age, language, cultural context, or training-system representation. The findings should therefore be interpreted within this specific 100-m freestyle race-day context. Second, only one standardized prompt and a single-turn procedure were used. Although this improved experimental control, it does not fully reflect real coaching practice, in which users may provide additional information, ask follow-up questions, correct recommendations, and iteratively refine outputs. The findings therefore represent first-response performance under standardized conditions and cannot determine the effects of alternative prompt designs, languages, or information granularity.

Third, the tools were evaluated through their official web interfaces on 1 March 2026. Because model versions and platform settings are updated frequently, the results should be viewed as a time-specific comparison rather than permanent rankings. Moreover, because the internal training-data composition and alignment processes of the evaluated systems were not examined in this study, the observed between-model differences cannot be attributed to specific sources of model or platform bias. The study also did not compare subscription costs, usage limits, regional availability, or differences between free and paid access tiers, which may influence the practical accessibility of these tools for coaches. Fourth, each tool generated only five outputs, so the stability findings are descriptive and exploratory rather than definitive estimates of reproducibility. Fifth, expert ratings reflected perceived physiological relevance and content quality, not actual performance, recovery, adherence, or injury outcomes. No real swimmers implemented the plans, and their real-world effectiveness remains untested. Finally, the domain-level findings should be interpreted cautiously, and the study-specific 40-item instrument requires further validation.

## 6 Conclusions

This study demonstrates that publicly accessible LLM-based AI tools can generate structured race-day warm-up and immediate post-race cool-down plans for a simulated 100-m freestyle swimmer. Their shared strength lies in rapidly organizing common coaching components into editable, table-based drafts with basic time, intensity, and exercise information. This indicates that AI can already support coaches by reducing part of the repetitive work involved in preparing preliminary plans, comparing alternatives, and communicating plan structure to athletes.

However, the four AI tools differed in their practical strengths and may therefore serve different coaching needs. In the present scenario, ChatGPT appeared most suitable when coaches needed a relatively complete and higher-quality draft that integrated structure, dose clarity, safety, and sport-specific relevance. Gemini may be useful when coaches require richer pre-race communication, psychological reminders, and detailed athlete-facing instructions, although its warm-up duration and total volume should be carefully screened. DeepSeek may be more suitable when coaches need additional individualization, environmental adaptation, and safety-related reminders, but its intensity ranges and recovery structure require expert checking. Doubao may be useful for quickly producing stable and concise templates, especially in situations where coaches need a consistent basic framework; however, its outputs require substantial revision because several sprint-swimming-specific details may be missing.

These findings suggest that AI tools should be used as coach-supervised drafting and comparison aids, not as stand-alone providers of swimming race-day plans. Coaches should generate multiple outputs, screen them against swimming-specific criteria, and revise the final plan according to the athlete’s event, schedule, condition, facility constraints, and safety needs. In this workflow, AI can improve planning efficiency, but expert coaches remain responsible for final decisions regarding intensity, sequencing, recovery, risk management, and individual adaptation. Future research should examine different strokes, race distances, athlete levels, prompt languages, and real-world coach–athlete implementation to determine how AI-assisted planning can more safely and effectively support competitive swimming practice.

## Data Availability

The original contributions presented in the study are included in the article/**Supplementary Material**. Further inquiries can be directed to the corresponding author.
