# Supplementary material for: Physiological relevance and generation-level stability of artificial intelligence-generated race-day warm-up and cool-down plans for sprint swimming: an expert-rated comparison of four large language models
Source: Front Physiol. 2026 Aug 27;17:1923231. doi: 10.3389/fphys.2026.1923231 (PMC13558089; doi:10.3389/fphys.2026.1923231)
Supplement: Supplementary file 1 [file Supplementaryfile1.docx]

**Supplementary Note 1. Standardized prompt used for AI-generated race-day plans**

**Warm-up prompt**

You are a swimming coach with extensive high-level competition experience. Develop a race-day warmup plan for a 16-year-old male 100-m freestyle swimmer at National Level II.

The athlete is 180cm tall and weighs 72 kg. The race starts at 09:00. The warm-up duration is 30 minutes, conducted in a 50-m pool, with access to a foam roller and a yoga mat. The aim is to enhance explosive performance and activate the neuromuscular system, and the plan must include both dryland and in-water warm-up components. Present the output in a table. For each component, provide a clearly specified dose (time, distance, number of sets, and rest interval). Intensity must be indicated using both an RPE scale (1-10) and the USA Swimming intensity-level classification.

**Cool-down prompt**

The athlete finishes the race at 09:10.

As the swimmer's coach, develop a scientifically grounded post-race cooldown plan. The total cool-down duration is 30 minutes, conducted in a

50-m pool, with access to a foam roller and a yoga mat. The aim is to facilitate a gradual down-regulation from the high-intensity race state, promote recovery, relieve local tightness and stiffness, and reduce the risk of delayed-onset muscle soreness, while avoiding excessive fatigue and preparing the athlete for subsequent training or the next race. Present the output in a table. For each component, provide a clearly specified dose (time, distance, number of sets, and rest interval). Intensity must be indicated using both an RPE scale (1-10) and the USA Swimming intensity-level classification.

**Supplementary Table S1: Full rating instrument**

**Pre-race Warm-up Plan Rating Instrument**

| **Dimension** | **Item No.** | **Rating item** | **Rating score** | | | | | **Comment** |
| --- | --- | --- | --- | --- | --- | --- | --- | --- |
|  |  |  | **1** | **2** | **3** | **4** | **5** | **Optional** |
| Scientific validity | 01 | **Does the plan content align with the physiological mechanisms and fundamental purposes of pre-race warm-up, rather than treating the warm-up as a mini training session?**  *(This item evaluates only whether the underlying principle is correct. It focuses on whether the plan reflects warm-up logic such as increased body temperature, neuromuscular activation, joint mobility preparation, and event-specific arousal; load precision and time appropriateness are evaluated in Item 02.)*  1 point: Clearly violates warm-up principles; the content is inappropriately training-oriented or contains factual errors.  2 points: Includes only a few warm-up elements, but the principles are confused or incompletely explained.  3 points: The principle is basically correct but too general, with key warm-up logic missing.  4 points: Reflects warm-up physiological logic relatively well, with only minor gaps.  5 points: Fully and accurately reflects warm-up principles; the purpose of pre-race warm-up is clear and logically coherent. |  |  |  |  |  |  |
|  | 02 | **Are intensity, total volume, and time allocation appropriate for achieving warm-up goals without inducing additional fatigue?**  *(This item evaluates only whether load configuration is appropriate. It focuses on whether the plan enables readiness without causing additional fatigue; the correctness of the underlying principle is not evaluated again.)*  1 point: The load is clearly inappropriate and may cause fatigue or insufficient activation.  2 points: Load distribution is imbalanced, and the time or total volume is not well arranged.  3 points: The load is generally acceptable, but rhythm, total volume, or time control remains ordinary.  4 points: Load and time allocation are relatively reasonable, with low fatigue risk.  5 points: Load control is precise and balances general warm-up, event-specific preparation, and fatigue control within 30 minutes. |  |  |  |  |  |  |
| Structural completeness | 03 | **Are the plan modules complete, covering both land-based and in-water warm-up, and do they progress from general to event-specific preparation?**  *(This item mainly evaluates whether key modules are included and whether the modules form a structural progression from lower to higher intensity and from general to event-specific content.)*  1 point: Key modules are missing and the structure is clearly incomplete.  2 points: Only some modules are covered, with weak progression.  3 points: The modules are basically complete, but transitions are ordinary.  4 points: The modules are relatively complete and the progression is clear.  5 points: The modules are complete with smooth transitions, forming a clear closed loop. |  |  |  |  |  |  |
|  | 04 | **Are the sequence and timing of each stage clear and aligned with the race-day process, such as check-in, waiting area, and pre-start preparation?**  *(This item mainly evaluates whether the process is executable on race day rather than detached from the actual competition timeline.)*  1 point: The sequence is disordered and clearly disconnected from the competition process.  2 points: A basic sequence is present, but timing connections are unreasonable.  3 points: Generally matches the competition process, but details are ordinary.  4 points: The sequence is clear and matches the race-day process relatively well.  5 points: The process is highly aligned with the competition timeline and can be executed smoothly. |  |  |  |  |  |  |
| Sport-specific targeting | 05 | **Does the content emphasize the short-duration, high-power-output preparation required for the 100-m freestyle, including high-frequency stroke-rhythm activation, speed-feel development, and rapid force-production readiness, rather than becoming endurance-oriented?**  *(This item focuses on whether the plan identifies the speed-rhythm-neural-arousal characteristics of the 100-m freestyle. If the content resembles a middle- or long-distance swimming warm-up or a general swimming warm-up, a lower score should be assigned.)*  1 point: Clearly generalized or endurance-oriented and not aligned with the demands of the 100-m freestyle.  2 points: Shows slight event-specific features but remains largely generalized.  3 points: Basically targets the 100-m freestyle, but event-specific stimulation is not prominent enough.  4 points: Has a clear event-specific direction and supports the 100-m freestyle race relatively well.  5 points: Highly aligned with the demands of the 100-m freestyle, with sufficient preparation for speed and rhythm. |  |  |  |  |  |  |
|  | 06 | **Does the plan cover preparation for key components of the 100-m freestyle, such as start reaction, first-15-m acceleration and underwater work, speed re-establishment after the turn, and final sprint rhythm?**  *(This item mainly evaluates whether the plan truly serves the competition context of this event rather than merely providing general swimming warm-up content.)*  1 point: Completely lacks preparation for key event-specific components.  2 points: Mentions these components only slightly, with clearly insufficient preparation.  3 points: Includes some event-specific components, but coverage is insufficient.  4 points: Covers key event-specific components relatively fully.  5 points: Provides complete key event-specific preparation that is highly aligned with the competition context. |  |  |  |  |  |  |
| Safety | 07 | **Does the plan include necessary risk warnings and precautions, such as management of pain or discomfort, hydration, injury-related restrictions, and abnormal on-site reactions?**  *(This item mainly evaluates whether the text reminds users of potential risks and responses rather than assuming that the athlete can complete all planned activities without difficulty.)*  1 point: Provides no risk warning and may mislead users.  2 points: Provides scattered reminders, but they are insufficient to ensure safety.  3 points: Provides basic reminders, but they are not specific or complete enough.  4 points: Provides relatively complete reminders that support safe implementation.  5 points: Provides sufficient and specific risk warnings, showing awareness of on-site contingency management. |  |  |  |  |  |  |
|  | 08 | **Are movement choices and stimulus arrangements within acceptable pre-race safety boundaries, avoiding inappropriate movements, excessive intensity, overly long high-speed segments, overly dense sprinting, or unnecessary risky arrangements?**  *(This item evaluates the safety of the plan itself. If arrangements may increase the risk of strain, fatigue, or loss of technical control, the score should be lowered.)*  1 point: Contains clear injury risks or inappropriate arrangements.  2 points: Contains certain risks and unclear safety boundaries.  3 points: Basically safe, but still has room for improvement.  4 points: Overall relatively safe, with no obvious inappropriate stimulus.  5 points: Movement and stimulus arrangements are cautious, with clear pre-race safety boundaries. |  |  |  |  |  |  |
| Individualization | 09 | **Does the plan clearly reflect individual and contextual information, including age 16, National Level II athlete, 100-m freestyle, 9:00 a.m. race, 30-minute warm-up, and 50-m pool?**  *(This item mainly evaluates whether the known background information is genuinely used for customization rather than mechanically repeated or applied as a template.)*  1 point: Completely template-based, with no reflection of individual or contextual information.  2 points: Only mechanically repeats background information, with insufficient customization.  3 points: Partly individualized, but the use of information is limited.  4 points: Clearly reflects the athlete and competition context.  5 points: Highly customized and fully uses all key information. |  |  |  |  |  |  |
|  | 10 | **Does the plan provide adjustment recommendations based on the athlete's condition on the day or changes in on-site constraints, such as suboptimal readiness, shortened available time, or limited facility access?**  *(This item mainly evaluates whether the plan is flexible and adjustable enough to accommodate race-day uncertainties.)*  1 point: Provides no adjustment recommendations and lacks flexibility.  2 points: Provides vague adjustment prompts, but they are weak in operability.  3 points: Provides a basic adjustment approach, but it is not specific enough.  4 points: Provides relatively practical adjustment options.  5 points: Provides specific and executable adjustment recommendations covering common on-site changes. |  |  |  |  |  |  |
| Practical applicability | 11 | **Does the plan provide directly executable parameters, such as time, distance, sets, repetitions, intervals, intensity, or rhythm cues?**  *(This item mainly evaluates whether the plan can be directly implemented by coaches or athletes. Plans containing only general principles without parameters should not receive high scores.)*  1 point: Basically cannot be directly implemented because key parameters are missing.  2 points: Parameters are seriously insufficient, making implementation difficult.  3 points: Can be roughly implemented, but details remain insufficient.  4 points: Parameters are relatively clear and the plan can basically be implemented directly.  5 points: Parameters are complete and clear, allowing direct implementation by coaches. |  |  |  |  |  |  |
|  | 12 | **Does the plan consider the availability of equipment and facilities and provide feasible alternatives, such as resistance bands, starting blocks, or changes in lane conditions?**  *(This item mainly evaluates real-world executability rather than whether the plan appears reasonable in theory but is difficult to implement at the competition venue.)*  1 point: Clearly detached from on-site conditions and provides no alternatives.  2 points: Partly considers on-site conditions, but feasibility is poor.  3 points: Basically feasible, but adaptation to facilities or equipment is ordinary.  4 points: Considers real-world conditions relatively well and provides alternatives.  5 points: Highly adapted to the competition venue, with clear and feasible alternatives. |  |  |  |  |  |  |
| Rationality of arrangement | 13 | **Is the overall pre-race regulation logic mature, improving physiological readiness and psychological race entry without adding extra training stimulus?**  *(This item evaluates whether pre-race regulation is appropriate and does not repeat the evaluation of principle correctness.)*  1 point: Clearly training-oriented, with inappropriate pre-race regulation.  2 points: The regulation approach is biased and may increase burden or distract attention.  3 points: Basically reasonable, but improvement in pre-race readiness is limited.  4 points: Balances activation, focus, and fatigue control relatively well.  5 points: The regulation logic is mature and can effectively improve pre-race physical and psychological readiness. |  |  |  |  |  |  |
|  | 14 | **Does the plan include concise and executable focus, breathing, or rhythm cues that help the athlete enter race mode without adding extra cognitive load?**  *(This item mainly evaluates whether pre-race regulation considers psychological state and attentional allocation rather than piling up complex instructions.)*  1 point: Provides no relevant cues, or the cues increase the burden.  2 points: Provides cues, but they are vague or impractical.  3 points: Basically usable, but ordinary.  4 points: Provides concise and feasible cues that help the athlete enter race mode.  5 points: Provides precise and light-touch cues that clearly support pre-race orientation and readiness. |  |  |  |  |  |  |
| Clarity and dose specification | 15 | **Is the language clear and easy to understand, are terms used appropriately, and are key instructions unambiguous?**  *(This item mainly evaluates whether users can accurately understand the meaning of the plan and avoid vague wording or mixed terminology.)*  1 point: The wording is disordered and difficult to understand.  2 points: Contains many ambiguities or inappropriate terms.  3 points: Basically understandable, but still not clear enough.  4 points: The wording is clear and terms are relatively appropriate.  5 points: The wording is very clear, standardized, and unambiguous. |  |  |  |  |  |  |
|  | 16 | **Are expressions of intensity, rhythm, sequence, and related instructions consistent throughout the plan, avoiding conflicting instructions within the same plan?**  *(This item mainly evaluates internal consistency rather than the elegance of individual sentences.)*  1 point: Contains clear contradictions and conflicting instructions.  2 points: Contains multiple inconsistencies that affect implementation.  3 points: Generally consistent, but some expressions are unstable.  4 points: Expressions are relatively unified, with few barriers to implementation.  5 points: Expressions of intensity, rhythm, and sequence are fully consistent and stable. |  |  |  |  |  |  |
| Innovation | 17 | **Does the plan provide reasonable highlights or individualized strategies without sacrificing safety and feasibility?**  *(This item does not reward novelty for its own sake; it rewards small optimizations that genuinely improve warm-up quality, such as better transitions, rhythm cues, or on-site adaptation.)*  1 point: Provides no highlight, or the so-called innovation is clearly unreasonable.  2 points: The highlight is forced and has limited value.  3 points: Shows some novelty, but the effect is ordinary.  4 points: Provides reasonable highlights that increase the value of the plan.  5 points: Provides clear, safe, and feasible highlights with genuine added value. |  |  |  |  |  |  |
|  | 18 | **Are innovative arrangements still consistent with event-specific goals and the competition context, rather than deviating from the core warm-up tasks of the 100-m freestyle?**  *(This item mainly evaluates the consistency between highlights and event-specific goals.)*  1 point: Innovation is clearly disconnected from the event-specific task.  2 points: Has a weak connection with event-specific goals.  3 points: Basically consistent, but integration is ordinary.  4 points: Serves event-specific goals and the competition context relatively well.  5 points: The highlights are highly aligned with and reinforce the warm-up objectives for the 100-m freestyle. |  |  |  |  |  |  |
| Overall quality | 19 | **After considering quality, risk, and executability, would you be willing to adopt this warm-up plan on race day?**  *(This item mainly evaluates overall willingness to use the plan and represents an integrated judgment across the preceding dimensions rather than a repetition of any single dimension.)*  1 point: Definitely would not adopt it.  2 points: Would consider using it only after substantial revision.  3 points: Could use it as a reference, but not directly.  4 points: Could adopt it after minor revision.  5 points: Basically willing to adopt it; it is close to being directly usable. |  |  |  |  |  |  |
|  | 20 | **If only minor revisions were allowed before use, how close is the plan to a version that could be directly adopted on race day?**  *(This item mainly evaluates how far the plan is from a practically usable version; a higher score indicates lower subsequent revision cost.)*  1 point: Very far from a usable version, with very high revision cost.  2 points: Still requires considerable rewriting and reorganization.  3 points: Requires a moderate level of revision before use.  4 points: Requires only minor revision before use.  5 points: Nearly a directly usable race-day version. |  |  |  |  |  |  |

**Immediate Post-race Cool-down Plan Rating Instrument**

| **Dimension** | **Item No.** | **Rating item** | **Rating score** | | | | | **Comment** |
| --- | --- | --- | --- | --- | --- | --- | --- | --- |
|  |  |  | **1** | **2** | **3** | **4** | **5** | **Optional** |
| Scientific validity | 01 | **Does the plan content align with the basic physiological principles of post-race recovery and cool-down, rather than treating the cool-down as continued training or additional stimulation?**  *(This item evaluates only whether the recovery principle is correct. It focuses on whether the plan reflects progressive heart-rate reduction, breathing stabilization, local muscle relaxation, subjective fatigue relief, and post-race recovery logic; load precision and time appropriateness are evaluated in Item 02.)*  1 point: Clearly violates recovery principles; the content is inappropriately training-oriented or contains factual errors.  2 points: Includes only a few recovery elements, but the principles are confused or incompletely explained.  3 points: The principle is basically correct but too general, with key recovery logic missing.  4 points: Reflects post-race recovery logic relatively well, with only minor gaps.  5 points: Fully and accurately reflects recovery principles; the purpose of post-race cool-down is clear and logically coherent. |  |  |  |  |  |  |
|  | 02 | **Are intensity, total volume, and time allocation appropriate for promoting heart-rate reduction and comfortable recovery without causing additional fatigue or renewed stimulation?**  *(This item evaluates only whether load configuration is appropriate. It focuses on whether the plan helps the body transition smoothly out of the race state without creating a new burden; the correctness of the underlying principle is not evaluated again.)*  1 point: The load is clearly inappropriate and may cause additional fatigue or insufficient recovery.  2 points: Load distribution is imbalanced, and the time or total volume is not well arranged.  3 points: The load is generally acceptable, but rhythm, total volume, or time control remains ordinary.  4 points: Load and time allocation are relatively reasonable, with low risk of additional stimulation.  5 points: Load control is precise and effectively promotes downregulation and comfortable recovery within limited time. |  |  |  |  |  |  |
| Structural completeness | 03 | **Are the plan modules complete, covering key components such as active downregulation, breathing/relaxation, local muscle relaxation or stretching, and basic post-race recovery reminders?**  *(This item mainly evaluates whether the post-race cool-down plan has a complete recovery chain rather than only including a brief easy swim or only stretching.)*  1 point: Key modules are missing and the structure is clearly incomplete.  2 points: Only some modules are covered, and the recovery chain is incomplete.  3 points: The modules are basically complete, but transitions are ordinary.  4 points: The modules are relatively complete and the recovery loop is clear.  5 points: The modules are complete with smooth transitions, forming a clear post-race recovery loop. |  |  |  |  |  |  |
|  | 04 | **Are the sequence of each stage and the completion criteria clear, and are they aligned with the actual post-race process, such as leaving the pool, organizing equipment, hydration, and subsequent recovery arrangements?**  *(This item mainly evaluates whether the process is executable on race day rather than detached from the actual competition timeline.)*  1 point: The sequence is disordered and clearly disconnected from the post-race process.  2 points: A basic sequence is present, but stage transitions are unreasonable.  3 points: Generally matches the post-race process, but details are ordinary.  4 points: The sequence is clear and matches the post-race process relatively well.  5 points: The process is highly aligned with the post-race timeline, can be executed smoothly, and has a clear ending. |  |  |  |  |  |  |
| Sport-specific targeting | 05 | **Does the content focus on the main post-race recovery needs of the 100-m freestyle, such as local muscle tension after high-intensity sprinting, breathing and heart-rate reduction, subjective fatigue relief, and reduced neural arousal?**  *(This item focuses on whether the plan identifies the high-intensity, short-duration, high-neural-arousal recovery characteristics after the 100-m freestyle. If the content resembles general post-training recovery or a generalized arrangement, a lower score should be assigned.)*  1 point: Clearly generalized and not aligned with the post-race demands of the 100-m freestyle.  2 points: Shows slight event-specific features but remains largely generalized.  3 points: Basically targets post-race recovery for the 100-m freestyle, but event-specific recovery priorities are not prominent enough.  4 points: Has a clear event-specific direction and supports post-race recovery for the 100-m freestyle relatively well.  5 points: Highly aligned with the post-race demands of the 100-m freestyle, with accurate identification of recovery priorities. |  |  |  |  |  |  |
|  | 06 | **Does the plan cover key post-race recovery components for the 100-m freestyle, such as local relaxation of the shoulders, back, hips, and legs, re-establishment of breathing rhythm, transition from short high-intensity stimulation, and connection with subsequent recovery?**  *(This item mainly evaluates whether the plan truly serves the post-race context of this event rather than providing generalized relaxation content.)*  1 point: Completely lacks key event-specific recovery components.  2 points: Mentions them only slightly, but coverage is clearly insufficient.  3 points: Includes some event-specific recovery components, but coverage is insufficient.  4 points: Covers key event-specific recovery components relatively fully.  5 points: Provides complete key event-specific recovery that is highly aligned with the post-race context of the 100-m freestyle. |  |  |  |  |  |  |
| Safety | 07 | **Does the plan include necessary risk warnings and precautions, such as management of dizziness, nausea, cramps, hydration, pain or injury-related restrictions, and abnormal reactions?**  *(This item mainly evaluates whether the text reminds users of potential risks and responses rather than assuming that the athlete can complete all planned activities without difficulty.)*  1 point: Provides no risk warning and may mislead users.  2 points: Provides scattered reminders, but they are insufficient to ensure safety.  3 points: Provides basic reminders, but they are not specific or complete enough.  4 points: Provides relatively complete reminders that support safe implementation.  5 points: Provides sufficient and specific risk warnings, showing awareness of post-race on-site contingency management. |  |  |  |  |  |  |
|  | 08 | **Are movement choices and stimulus arrangements within acceptable post-race safety boundaries, avoiding secondary high-intensity stimulation, overstretching, exercising through pain, or unnecessary risky arrangements?**  *(This item evaluates the safety of the plan itself. If arrangements may increase the risk of strain, cramping, loss of technical control, or delayed recovery, the score should be lowered.)*  1 point: Contains clear injury risks or inappropriate arrangements.  2 points: Contains certain risks and unclear safety boundaries.  3 points: Basically safe, but still has room for improvement.  4 points: Overall relatively safe, with no obvious inappropriate stimulus.  5 points: Movement and stimulus arrangements are cautious, with clear post-race safety boundaries. |  |  |  |  |  |  |
| Individualization | 09 | **Does the plan clearly reflect individual and contextual information, including age 16, National Level II athlete, 100-m freestyle, immediate post-race context, 50-m pool, and available equipment and facility conditions?**  *(This item mainly evaluates whether the known background information is genuinely used for customization rather than mechanically repeated or applied as a template.)*  1 point: Completely template-based, with no reflection of individual or contextual information.  2 points: Only mechanically repeats background information, with insufficient customization.  3 points: Partly individualized, but the use of information is limited.  4 points: Clearly reflects the athlete and competition context.  5 points: Highly customized and fully uses all key information. |  |  |  |  |  |  |
|  | 10 | **Does the plan provide adjustment recommendations based on post-race status or on-site constraints, such as heavy fatigue, discomfort, shortened available time, or limited facility access?**  *(This item mainly evaluates whether the plan is flexible and adjustable enough to accommodate post-race uncertainties.)*  1 point: Provides no adjustment recommendations and lacks flexibility.  2 points: Provides vague adjustment prompts, but they are weak in operability.  3 points: Provides a basic adjustment approach, but it is not specific enough.  4 points: Provides relatively practical adjustment options.  5 points: Provides specific and executable adjustment recommendations covering common post-race changes. |  |  |  |  |  |  |
| Practical applicability | 11 | **Does the plan provide directly executable parameters, such as time, distance, sets, repetitions, intervals, intensity, or rhythm cues?**  *(This item mainly evaluates whether the plan can be directly implemented by coaches or athletes. Plans containing only general principles without parameters should not receive high scores.)*  1 point: Basically cannot be directly implemented because key parameters are missing.  2 points: Parameters are seriously insufficient, making implementation difficult.  3 points: Can be roughly implemented, but details remain insufficient.  4 points: Parameters are relatively clear and the plan can basically be implemented directly.  5 points: Parameters are complete and clear, allowing direct implementation by coaches. |  |  |  |  |  |  |
|  | 12 | **Does the plan consider the availability of equipment and facilities and provide feasible alternatives, such as kickboards, foam rollers, stretching areas, lane restrictions, or changes in recovery conditions after leaving the pool?**  *(This item mainly evaluates real-world executability rather than whether the plan appears reasonable in theory but is difficult to implement at the competition venue.)*  1 point: Clearly detached from on-site conditions and provides no alternatives.  2 points: Partly considers on-site conditions, but feasibility is poor.  3 points: Basically feasible, but adaptation to facilities or equipment is ordinary.  4 points: Considers real-world conditions relatively well and provides alternatives.  5 points: Highly adapted to the post-race setting, with clear and feasible alternatives. |  |  |  |  |  |  |
| Rationality of arrangement | 13 | **Is the overall post-race regulation logic mature, promoting physiological downregulation, comfortable subjective recovery, and transition to subsequent recovery without adding training stimulus?**  *(This item evaluates whether post-race regulation is appropriate and does not repeat the evaluation of principle correctness.)*  1 point: Clearly training-oriented, with inappropriate post-race regulation.  2 points: The regulation approach is biased and may increase burden or delay recovery.  3 points: Basically reasonable, but its contribution to recovery is limited.  4 points: Balances downregulation, relaxation, and transition to subsequent recovery relatively well.  5 points: The regulation logic is mature and can effectively promote post-race physical and psychological recovery. |  |  |  |  |  |  |
|  | 14 | **Does the plan avoid describing any single recovery method as inevitably and substantially effective, while reflecting individual differences, recovery options, and evidence boundaries?**  *(This item mainly evaluates whether recovery strategies are expressed scientifically and cautiously without overstating the definite effect of any single method.)*  1 point: Contains clear overstatements or misleading claims.  2 points: Shows a tendency toward exaggeration, with unclear boundaries.  3 points: Basically cautious, but some wording is still too strong.  4 points: Expressed relatively cautiously and reflects differences in recovery responses.  5 points: Expressed scientifically and conservatively, fully reflecting individual differences and evidence boundaries. |  |  |  |  |  |  |
| Clarity and dose specification | 15 | **Is the language clear and easy to understand, are terms used appropriately, and are key instructions unambiguous?**  *(This item mainly evaluates whether users can accurately understand the meaning of the plan and avoid vague wording or mixed terminology.)*  1 point: The wording is disordered and difficult to understand.  2 points: Contains many ambiguities or inappropriate terms.  3 points: Basically understandable, but still not clear enough.  4 points: The wording is clear and terms are relatively appropriate.  5 points: The wording is very clear, standardized, and unambiguous. |  |  |  |  |  |  |
|  | 16 | **Are expressions of intensity, rhythm, sequence, and related instructions consistent throughout the plan, avoiding conflicting instructions within the same plan?**  *(This item mainly evaluates internal consistency rather than the elegance of individual sentences.)*  1 point: Contains clear contradictions and conflicting instructions.  2 points: Contains multiple inconsistencies that affect implementation.  3 points: Generally consistent, but some expressions are unstable.  4 points: Expressions are relatively unified, with few barriers to implementation.  5 points: Expressions of intensity, rhythm, and sequence are fully consistent and stable. |  |  |  |  |  |  |
| Innovation | 17 | **Does the plan provide reasonable highlights or beneficial recovery strategies without sacrificing safety and feasibility?**  *(This item does not reward novelty for its own sake; it rewards small optimizations that genuinely improve post-race cool-down quality, such as better transition design, low-burden breathing relaxation, concise recovery reminders, or on-site adaptation strategies.)*  1 point: Provides no highlight, or the so-called innovation is clearly unreasonable.  2 points: The highlight is forced and has limited value.  3 points: Shows some novelty, but the effect is ordinary.  4 points: Provides reasonable highlights that increase the value of the plan.  5 points: Provides clear, safe, and feasible highlights with genuine added value. |  |  |  |  |  |  |
|  | 18 | **Are innovative arrangements still consistent with event-specific goals and the competition context, rather than deviating from the core post-race cool-down tasks of the 100-m freestyle?**  *(This item mainly evaluates the consistency between highlights and recovery goals.)*  1 point: Innovation is clearly disconnected from the event-specific recovery task.  2 points: Has a weak connection with event-specific recovery goals.  3 points: Basically consistent, but integration is ordinary.  4 points: Serves event-specific goals and the post-race context relatively well.  5 points: The highlights are highly aligned with and reinforce the post-race recovery/cool-down objectives for the 100-m freestyle. |  |  |  |  |  |  |
| Overall quality | 19 | **After considering quality, risk, and executability, would you be willing to adopt this cool-down plan on race day?**  *(This item mainly evaluates overall willingness to use the plan and represents an integrated judgment across the preceding dimensions rather than a repetition of any single dimension.)*  1 point: Definitely would not adopt it.  2 points: Would consider using it only after substantial revision.  3 points: Could use it as a reference, but not directly.  4 points: Could adopt it after minor revision.  5 points: Basically willing to adopt it; it is close to being directly usable. |  |  |  |  |  |  |
|  | 20 | **If only minor revisions were allowed before use, how close is the plan to a version that could be directly adopted after the race?**  *(This item mainly evaluates how far the plan is from a practically usable version; a higher score indicates lower subsequent revision cost.)*  1 point: Very far from a usable version, with very high revision cost.  2 points: Still requires considerable rewriting and reorganization.  3 points: Requires a moderate level of revision before use.  4 points: Requires only minor revision before use.  5 points: Nearly a directly usable post-race version. |  |  |  |  |  |  |

**Table S2. Inter-rater reliability of aggregated expert ratings**

| **Outcome** | **Measure** | **ICC** | **95% CI** | **F** | **df1** | **df2** | **p** |
| --- | --- | --- | --- | --- | --- | --- | --- |
| Warm-up mean score | Single-measure ICC | 0.801 | 0.690–0.898 | 201.518 | 19 | 589 | <0.001 |
|  | Average-measure ICC | 0.992 | 0.986–0.996 | 201.518 | 19 | 589 | <0.001 |
| Cool-down mean score | Single-measure ICC | 0.660 | 0.506–0.813 | 141.522 | 19 | 589 | <0.001 |
|  | Average-measure ICC | 0.984 | 0.970–0.993 | 141.522 | 19 | 589 | <0.001 |
| Overall mean score | Single-measure ICC | 0.770 | 0.639–0.882 | 244.571 | 19 | 589 | <0.001 |
|  | Average-measure ICC | 0.991 | 0.983–0.996 | 244.571 | 19 | 589 | <0.001 |

**Note.** ICC = intraclass correlation coefficient; CI = confidence interval. Single-measure ICC reflects the reliability of ratings from an individual expert, whereas average-measure ICC reflects the reliability of the mean rating across all 32 experts. ICCs were calculated using a two-way random-effects, absolute-agreement model. Warm-up, cool-down, and overall scores were calculated as mean scores across the corresponding rating items.

**Table S3. Krippendorff’s alpha for item-level ordinal expert ratings**

| **Outcome** | **Krippendorff’s alpha (α)** | **Scale** | **Rating** |
| --- | --- | --- | --- |
| Warm-up item-level ratings | 0.559 | Ordinal | 1–5 Likert scale |
| Cool-down item-level ratings | 0.536 | Ordinal | 1–5 Likert scale |
| Overall item-level ratings | 0.548 | Ordinal | 1–5 Likert scale |

**Note.** α = Krippendorff’s alpha. Krippendorff’s alpha was calculated using an ordinal metric for item-level 1–5 Likert ratings. Warm-up and cool-down analyses included the corresponding section-specific item-level ratings, whereas the overall analysis included all item-level ratings across both sections. These values reflect inter-rater agreement at the single-item level rather than the reliability of aggregated mean scores.
